# Bioelectrochemical systems and synthetic biology: more power, more products

**DOI:** 10.1111/1751-7915.13456

**Published:** 2019-07-01

**Authors:** Sarah M. Glaven

**Affiliations:** ^1^ Center for Bio/Molecular Science and Engineering Naval Research Laboratory Washington DC USA

Microbial electrochemical technologies (METs) are applications or processes that utilize the electrochemical interaction of microbes and electrodes (Schroder *et al*., 2015). It has been known for over 100 years (Potter, 1911) that microorganisms can form electrical connections to devices, but only recently (approximately 20 years) has this concept been put to technological use. Microbial electrochemistry and electromicrobiology have grown as disciplines due to an intense interest in the possibility of using MET for alternative energy, wastewater treatment and biofuels production. METs have the potential to contribute to a circular economy, where carbon is cycled back into products or electricity from renewable sources.

The natural physiological activity of electroactive bacteria, those capable of extracellular electron transfer (EET), has been studied intensely over the past two decades in order to improve efficiency and productivity of METs. A diverse group of scientists has contributed to this knowledge base including microbiologists, electrochemists, physicists, biochemists and molecular biologists. Genetic engineering has been used to determine the molecular underpinnings responsible for carrying charge between cells and the electrode of the two model EET organisms, *Geobacter sulfurreducens* and *Shewanella oneidensis*. New electroactive organisms have been discovered using genomics (Eddie *et al*., 2016), and new ways to transform electrode‐associated microbial communities are being developed. Electrochemistry, advanced imaging techniques and modelling have all been employed to track the movement of electrons through biofilms and purified proteins. New lexicons have been created to allow interdisciplinary discussions of extracellular electron transfer, and an entire Center for Electromicrobiology has been funded at Aarhus University in Denmark.

Our growing knowledge of the principles of EET is now poised to intersect with the nascent field of synthetic biology to bring about the next generation of MET for power and energy, microbial electrosynthesis and microbial bioelectronics. Synthetic biology is an emerging field that has grown out of the principles of biology and engineering disciplines, devoted to the rational design and engineering of organisms and their components (Church *et al*., 2014). It is now possible to both engineer specific functions into living systems and construct entirely new ones (Liu *et al*., 2018a,b). Microorganisms are viewed as tiny supercomputers that can be programmed as such by reading, writing and editing the cell's DNA. Genetic circuits can be designed computationally to wire cells for on‐demand functionality (Nielsen *et al*., 2016), and these circuits can be printed and shipped to a scientist at the bench to deploy in the organism of their choice (Libby and Silver, 2019). Growth of the field of synthetic biology has resulted in the development of new tools and approaches in molecular genetics to advance biotechnology across a wide application space. For example, it is now possible to precisely tune gene expression from 12 independent small‐molecule sensors engineered into the genome of *E. coli* (Meyer *et al*., 2018) or confer the ability of a bacterial cell to ‘see’ different wavelengths of light and respond in a pre‐programmed manner (Fernandez‐Rodriguez *et al*., 2017). Synthetic biology has the potential to advance microbial electrochemical technologies (MET) by bringing new design platforms to engineer EET pathways in organisms that do not naturally have them, modify microbial metabolism to improve EET rates and increase the diversity of products from microbial electrosynthesis. Two examples are given below: (i) enhanced power output from microbial fuel cells and (ii) the potential for microbial electrosynthesis to be a viable approach for fuels or molecules production.

## Power and energy – microbial fuel cells

Despite nearly two decades of research, microbial fuel cells (MFC) are still considered a nascent area of research. One of the first and most well‐known examples of a viable technology from these bacterial batteries is the benthic microbial fuel cell (BMFC). In a BMFC, energy is harvested from the seafloor to power oceanographic sensors (Tender *et al*., 2008) using microorganisms that naturally colonize the anode electrode to catalyse the conversion of organic carbon to electrons. BMFCs have shown that free, persistent power can be generated from organic matter in the sediment, replacing the need for rechargeable batteries on sensors and devices in the ocean. Progress has been made in increasing the power output of MFCs simply by exploiting natural microbial communities found in environments such as benthic sediment, hydrothermal vents, domestic wastewater and industrial wastewater. Some small start‐ups have overcome some cost barriers and are developing viable commercial technologies, including Aquacycl and iMETland, which are using MFCs to generate electricity from wastewater through small, scalable modular units or constructed wetlands, respectively. Also promising is the direct use of urine to power a self‐sufficient lit urinal system (Walter *et al*., 2018).

While newly installed wind and solar energy sources are generating electricity at a cost that is competitive with fossil fuels (10 cents kWh^−1^) (Lazard, 2016), the capital costs associated with MFC technology are still high compared with power output. An analysis of the economic viability of energy from MFCs has been attempted primarily using wastewater as a feedstock (Stoll *et al*., 2016; Trapero *et al*., 2017). Cost comparisons are challenging due to the wide range in reported bioelectrochemical system (BES) designs (Zhang and Angelidaki, 2016). While miniaturized MFCs with high power density have been demonstrated, scaling decreases power output generally below 1 W m^−3^ (Dong *et al*., 2015), and the potential revenue from electricity production is not cost‐attractive from an energy production standpoint (Stoll *et al*., 2016).

If material and design costs could be reduced, the primary factors which limit power overall are as follows: (i) the catalytic rate of the oxygen reduction reaction at the cathode, (ii) diffusion of substrate to or products from the electrodes and (iii) conductivity and buffering capacity of the electrolyte (Popat *et al*., 2014; Popat and Torres, 2016; Logan *et al*., 2018; Rossi *et al*., 2019). While the latter two are largely system engineering problems, synthetic biology could be used to help create new catalysts for the cathode reaction by either employing living microorganisms or using microorganisms to biologically produce a new catalytic material itself. A recent workshop on synthetic biology for use in power and energy summarized the major impacts of synthetic biology on energy‐related projects and potential for catalyst development (Jewett *et al*., 2018).

Ultimately, the microbial turnover of organic substrate (feedstock), and subsequent diffusion of electrons to the electrode surface, will dictate the number of electrons liberated to the anode electrode for electricity generation. If an infinite supply of electrons were available from the cell, then surely opening the valve and letting more out would be the answer to making more electricity and driving power production (Izallalen *et al*., 2008; Feist *et al*., 2014). The problem is that bacteria have many ways to balance their electrons and many redundancies built into EET which we are only now beginning to understand. Flux‐balance modelling coupled to genetic engineering and synthetic biology could improve rate‐limiting steps, and increasing the number of biofilm‐bound charge carriers will improve diffusion rate of electrons. Key to employing this strategy will be defining the rules that govern the cell's ability to manufacture and deploy charge‐carrying proteins. Successful attempts at expressing the Mtr pathways from *S. oneidensis* in *E. coli* have proven that it is possible to engineer EET into chassis strains (Jensen *et al*., 2010). Improvements to these designs now largely depend on development of high‐throughput (HTP) electrochemical approaches to screen combinatorial libraries of EET proteins for improved functionality.

## Microbial electrosynthesis

Following the realization that microorganisms drive electricity production at the anode electrode, it was also determined that electrons could run in reverse and provide reducing equivalents to drive reduction reactions. Initially, the possibility to drive reductive decontamination of groundwater contaminants was tested (Gregory *et al*., 2004; Strycharz *et al*., 2008); however, it was quickly realized that reduction of CO_2_ into fuels, a process termed microbial electrosynthesis, could be a more viable technology (Nevin *et al*., 2010; Rabaey and Rozendal, 2010). Microbial electrosynthesis, where microorganisms are employed to convert CO_2_ to a range of useful products by behaving as catalysts for conversion of electrons, received directly or via electron transfer mediators into stable bonds of organic chemicals. For example, microbial electrosynthesis of acetate from CO_2_ and electrons has been demonstrated using both pure cultures and mixed microbial communities and has generally been dependent on hydrogen generation at the electrode where hydrogen serves as an electron transfer mediator to cells (Nevin *et al*., 2010; Marshall *et al*., 2013, 2017; LaBelle and May, 2017). More recently, interesting progress has been made to develop photosynthetic autotrophs for microbial electrosynthesis applications (Guzman *et al*., 2019).

For purely electrochemical conversion of CO_2_ to biofuels such as ethanol, a recent technoeconomic analysis indicates that this manner of conversion is still not cost‐competitive with existing commercial prices, primarily due to the cost of the electrolyser units (Spurgeon and Kumar, 2018). Although introduction of microorganisms does not necessarily improve the overall cost analysis of chemical electrosynthesis, it provides an opportunity to exploit microorganisms to introduce electrosynthesis into new product markets. Technoeconomic analysis of acetate production from MES has recently been performed (Christodoulou and Velasquez‐Orta, 2016), and it was found that, just as for MFCs, operating costs are the primary factor holding back this technology as costs remain high compared with conventional methods for acetate production (33% higher than the commercial acetic acid price in the UK), including methanol carbonylation and ethane direct oxidation, which are at least 1.8 times lower costs than the commercial price of acetate. Synthetic biology could help reduce these costs by converting CO_2_ to higher value chemicals and potentially improving the biocatalyst itself to increase production rates and high reaction specificity which is not achievable using conventional metal and chemical catalysts.

Molecules production using MES is an area in which synthetic biology could make a major impact due to the ability to rapidly design and transform relevant chassis organisms for chemical production (Casini *et al*., 2018). This was recently demonstrated by a team of researchers at the MIT/Broad Institute as part of a DARPA pressure test for chemical synthesis to rapidly meet the needs of various sectors of the U.S. and global economy and defence sector (Casini *et al*., 2018). Renewable energy, such as solar, can be used to drive water‐splitting reactions to provide electrons to the microbial catalyst (Liu *et al*., 2018a,b) further reducing operating costs.

Another MET that could be impacted by synthetic biology tools for molecule production is known as electrofermentation – the guided fermentation of waste or traditional feedstock using an electrode (Flynn *et al*., 2010; Christodoulou and Velasquez‐Orta, 2016). Other than improvements to product formation, MES could be enhanced by engineering the pathways that electrons travel into cells and their energy‐coupling reactions (Tefft and TerAvest, 2019).

## Engineered conductive materials and bioelectronic technologies

The use of synthetic biology tools and platforms to improve MFCs and MES will also help grow areas of electromicrobiology and microbial electrochemical technologies that are just beginning to arise. Efforts to develop electrical reporting devices have continued to improve (Jensen *et al*., 2010, 2016; Goldbeck *et al*., 2013; West *et al*., 2017) and are now moving from *E. coli* to organisms tolerant to conditions outside of the laboratory (Bird *et al*., 2019). Synthetic biology is being used to allow for faster reporting using EET pathways by refactoring split ferredoxins as switchable gates for electrons (Atkinson *et al*., 2019). In addition, it was recently shown that redox active molecules can be used to turn on gene expression in *E. coli* depending on regeneration of the molecule using an electrode (Tschirhart *et al*., 2017).

In the future, microorganisms will be used to build self‐healing, self‐replicating living conductive materials. Such materials could replace traditional electronics in environmental sensing coupled to such technologies as bioremediation. Electromicrobiology will be used to directly connect the human microbiome, either the skin or the gut microbiome, to devices useful for personalized medicine or monitoring (Light *et al*., 2018). We know that the body is covered with bacteria. We should be using electronic devices to talk to them and for them to talk to us. METs can translate the language of microbes into our own.

## Concluding remarks

Synthetic biology could have near term impacts on the economic feasibility of MFCs and MES by improving power yields and product range, respectively. In the mid and long term, synthetic biology with enable bioelectronics and engineered living materials to become a reality. To implement the use of synthetic biology for BES, we need three things (i) high‐throughput (HTP) electrochemical screening technologies, (ii) an integrated data workflow to combine output from HTP electrochemical assays with genetic constructs, variants and combinatorial libraries and (iii) a curated, searchable database of electrochemically active (EA) organisms. In addition, we need to advance our understanding of the fundamental design rules on expressing components of EET in cells, or secreted from cells. Researchers are poised to make exponential improvements in the development of these technologies as data analysis and engineering tools improve.

## Conflict of Interest

None declared.
